# Microenvironment regulates the expression of miR-21 and tumor suppressor genes PTEN, PIAS3 and PDCD4 through ZAP-70 in chronic lymphocytic leukemia

**DOI:** 10.1038/s41598-017-12135-7

**Published:** 2017-09-25

**Authors:** Júlia Carabia, Cecilia Carpio, Pau Abrisqueta, Isabel Jiménez, Noelia Purroy, Eva Calpe, Carles Palacio, Francesc Bosch, Marta Crespo

**Affiliations:** Laboratory of Experimental Hematology, Department of Hematology, Vall d’Hebron Institute of Oncology, Vall d’Hebron University Hospital, Universitat Autònoma de Barcelona, Barcelona, Spain

## Abstract

Chronic lymphocytic leukemia (CLL) cells are highly dependent on microenvironment, being the BCR pathway one key player in this crosstalk. Among proteins participating, ZAP-70 enhances response to microenvironmental stimuli. MicroRNA-21 (miR-21) is overexpressed in diverse neoplasias including CLL, where it has been associated to refractoriness to fludarabine and to shorter time to progression and survival. To further elucidate the role of ZAP-70 in the biology of CLL, we studied its involvement in miR-21 regulation. MiR-21 expression was higher in CLL cells with high ZAP-70. Ectopic expression of ZAP-70 induced transcription of miR-21 via MAPK and STAT3, which subsequently induced downregulation of tumor suppressors targeted by miR-21. The co-culture of primary CLL cells mimicking the microenvironment induced ZAP-70 and miR-21 expression, as well as downregulation of miR-21 targets. Interestingly, the increase in miR-21 after co-culture was significantly impaired by ibrutinib, indicating that the BCR signaling pathway is involved in its regulation. Finally, survival of CLL cells induced by the co-culture correlated with miR-21 upregulation. In conclusion, stimuli from the microenvironment regulate miR-21 and its targeted tumor suppressor genes via a signaling pathway involving ZAP-70, thus contributing to the cytoprotection offered by the microenvironment particularly observed in CLL cells expressing ZAP-70.

## Introduction

Microenvironment found in bone marrow and lymph nodes supports survival, proliferation and drug resistance in chronic lymphocytic leukemia (CLL)^1^. Indeed, CLL cells are highly dependent on interactions with the microenvironment, as evidenced by the spontaneous apoptosis occurring *ex vivo* and the clinical relevance of impeding the access of CLL cells to lymph nodes and bone marrow that targeted treatments such as BTK or PI3K inhibitors induce^2^. The B-cell receptor (BCR) is one of the key players involved in the crosstalk between CLL cells and the microenvironment with a critical role in pathogenesis and prognosis in CLL. Accordingly, different factors related to increased signaling in the BCR pathway, such as unmutated IGHV genes^3^, high expression of ZAP-70 protein^4^, or increased serum levels of CCL3^5^ are associated with an adverse prognosis in CLL. Expression of ZAP-70 in CLL cells has been related to enhanced response to BCR stimulation, as well as to increased response to diverse migrative and survival stimuli from the microenvironment^6^. Moreover, we recently described how mimicking the microenvironment *ex vivo* leads to the upregulation of ZAP-70 protein in primary CLL cells and how the small percentage of Ki-67 positive cells in peripheral blood (PB) of patients with ZAP-70 are highly enriched in CLL cells expressing ZAP-70^7^.

Aberrant expression of diverse microRNAs (miRNAs) has been related to pathogenesis and clinical outcome in patients diagnosed with CLL (reviewed in ref.^8^). Among them, miR-21 is overexpressed in a wide variety of tumors, where it participates in different oncogenic processes^9^ and has been associated with poor prognosis^10^. Interestingly, overexpression of miR-21 in mice leads to a pre-B malignant lymphoid-like phenotype, demonstrating that miR-21 is a genuine oncogene^11^. Also, miR-21 has been shown to be overexpressed in activated B cells, particularly in germinal center and memory B cells. Its expression can be induced by IL-4, IL-4 and CD40L, or by BCR stimulation, suggesting that it may help to maintain B-cell hyperactivation which would prime B cells for malignant transformation^12^.

In CLL, miR-21 is overexpressed^13,14^ and it has been associated to refractoriness to fludarabine^15^, shorter overall survival and higher probability of progression^16^. Against this background, and in order to further elucidate the role of ZAP-70 in the crosstalk between CLL cells and the microenvironment, we studied the potential relationship between ZAP-70 protein and miR-21 and how it would be influenced by the microenvironment. Herein we define the correlation and participation of ZAP-70 protein in the regulation of miR-21 expression. Briefly, we have showed that stimuli from the microenvironment are capable of regulating the expression of miR-21 and its targeted tumor suppressor genes (PTEN, PDCD4 and PIAS3) via a signaling pathway involving ZAP-70, MAPK and STAT3 transcription factor, which correlates with the induction of survival by the microenvironment. These results help to enlighten the biology behind the adverse clinical outcome of patients with CLL and high expression of ZAP-70 and describe the participation of miR-21 in the crosstalk between CLL cells and the microenvironment.

## Results

### Expression of microRNA miR-21 is significantly higher in CLL cells with high expression of ZAP-70

**Figure 1:**
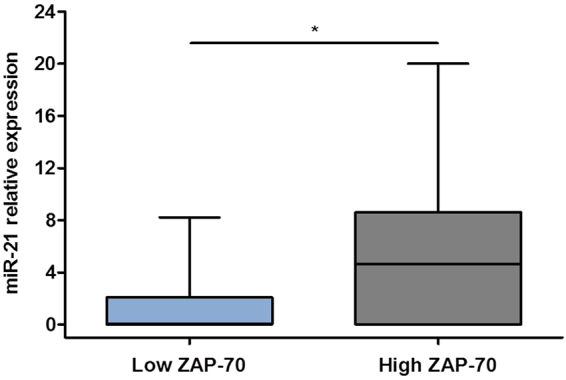
Expression of miR-21 is significantly higher in patients with CLL with high expression of ZAP-70. Expression levels of miR-21 were measured by QRT-PCR in PBMC from 33 patients diagnosed with CLL with high ZAP-70 (N = 17) or low ZAP-70 (N = 16), as indicated at the bottom of the histogram. *P < 0.05.

The relationship of miR-21 expression with indicators of bad prognosis in CLL and its role in B lymphocytes activation and oncogenesis prompted us to initially analyze the differential expression of miR-21 according to ZAP-70 status in primary cells from patients diagnosed with CLL. For that we purified CD19+ cells by means of magnetic separation from peripheral blood mononuclear cells (PBMC) in 33 patients diagnosed with CLL with either high (N = 17) or low (N = 16) expression of ZAP-70 as assessed by flow cytometry. Purity, further determined by flow cytometry, was superior to 90% in all cases. We then determined the expression of miR-21 by QRT-PCR and found that it was significantly higher in patients with high expression of ZAP-70 (mean miR-21 expression: 5.781 ± 1.517) than in patients with low ZAP-70 expression (mean miR-21 expression: 1.149 ± 0.5355; p = 0.0183) (Fig. 1). The main characteristics of the patients are detailed in Table 1.

**Table 1:** Characteristics of the patients. Data are expressed as median (Q1–Q3) or in percentages. M: Mutated; UM: Unmutated; N: Negative; P: Positive.

Gender (F/M in %)	65/35
Age (years)	65 (56–76)
17p deletion (N/P in %)	86 /14
13q deletion (N/P in %)	41/59
11q deletion (N/P in %)	84/16
Complex karyotype (No/Yes in %)	90/10
ZAP-70 categorized (Low/High in %)	56/44
IGHV (UM/M in %)	54/46
CLL cells (% CD19/CD5+)	87 (80–94)

### In B cells, ZAP-70 protein enhances the induction of expression of miR-21 after BCR stimulation via MAPK and STAT3 activation

**Figure 2:**
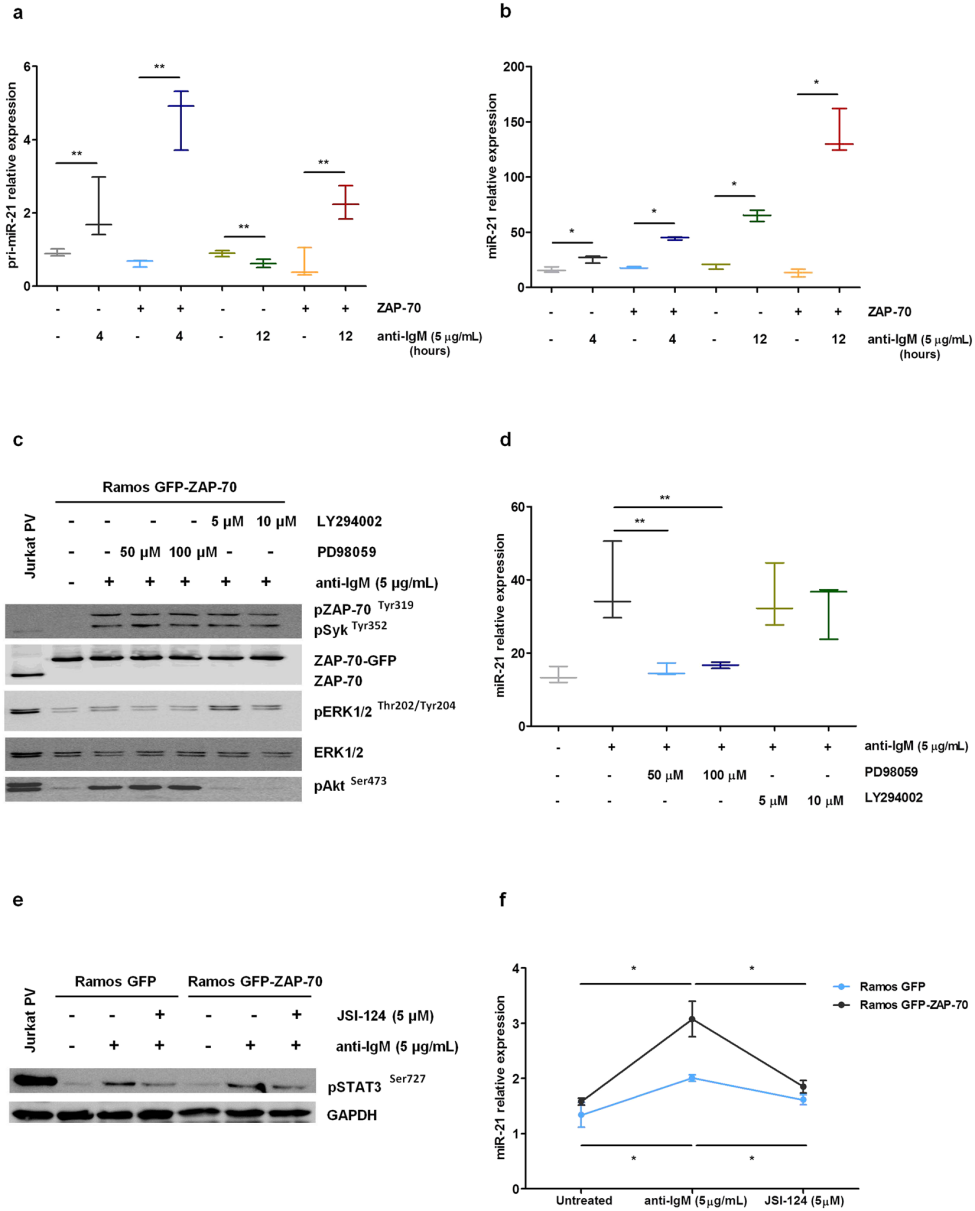
ZAP-70 protein overexpression enhances the induction of miR-21 expression after BCR stimulation in B cells via MAPK and STAT3 activation. (**a**–**b**) Ramos stable transfectants were stimulated with 5 µg/mL F(ab’)_2_ anti-IgM for 4 and 12 hours. Expression levels of primary miR-21 (**a**) and miR-21 (**b**) were measured by QRT-PCR. (**c**–**d**) Ramos GFP-ZAP-70 cells were stimulated with 5 µg/mL F(ab’)_2_ anti-IgM for 5 minutes after pre-incubation for 1 hour with 5 µM or 10 µM LY294002 for Akt inhibition or 50 µM or 100 µM PD98059 for ERK1/2 inhibition. Immunobloting analysis confirmed the inhibition of phosphorylation of Akt and ERK1/2 (Jurkat cells treated with PV were used as positive control) (**c**) and expression levels of miR-21 were measured by QRT-PCR after 12 hours (**d**). (**e**–**f**) Ramos stable transfectants were stimulated with 5 µg/mL F(ab’)_2_ anti-IgM for 5 minutes and pre-treated 1 hour with 5 µM JSI-124 for STAT3 inhibition. Immunobloting analysis confirmed the inhibition of STAT3 phosphorylation (Jurkat cells treated with PV were used as positive control) (**e**) and expression levels of miR-21 were measured by QRT-PCR after 48 hours (**f**). *P < 0.05, **P < 0.005.

ZAP-70 protein participates in different signaling pathways that modulate the interaction of CLL cells with the microenvironment. To elucidate if ZAP-70 protein could be directly involved in miR-21 upregulation in CLL, we stimulated the BCR of Ramos malignant B-cells stably transfected with a vector expressing a GFP-ZAP-70 fusion protein or GFP-only as a control. After 4 hours of anti-IgM stimulation we observed a 2.217 fold increase of the primary transcript of miR-21 in control Ramos cells (from 0.911 AU ± 0.055 to 2.019 AU ± 0.487), whereas in Ramos-ZAP-70 this fold increase went up to 7.387 (from 0.63 AU ± 0.057 to 4.654 AU ± 0.484) (Fig. 2a). After 12 hours, however, the increase was smaller and only observed in ZAP-70 expressing cells. On the contrary, mature miR-21 expression was only slightly increased after 4 hours of BCR stimulation (1.619 fold increase in control Ramos cells, and 2.485 fold increase in Ramos GFP-ZAP-70 cells), whereas after 12 hours the increase in expression was much higher, especially in cells expressing ZAP-70 (10.462 fold increase) (Fig. 2b). These results are in agreement with a regulation of miR-21 at the transcriptional level, since we observe a fast increment in its primary transcript followed by an increase in mature miR-21 levels after processing. Therefore, the results indicate that ZAP-70 activation after BCR signaling pathway stimulation is involved in the induction of the transcription of the primary transcript of miR-21 and subsequent increase in mature miR-21 levels.

In order to dissect the signaling pathway involved in the upregulation of miR-21 after ZAP-70 activation via BCR crosslinking we inhibited for 1 hour both Akt and ERK1/2 kinases using LY294002 (5 µM or 10 µM) or PD98059 (50 µM or 100 µM), respectively, in Ramos GFP-ZAP-70 B cells. In this system, the inhibition of ERK1/2 kinase completely abrogated the rise in miR-21 observed after BCR stimulation, whereas Akt inhibition had no effect (Fig. 2c and d).

The stimulation of the BCR signaling pathway leads to the nuclear translocation and activation of different transcription factors, including STAT3, which binds to the promoter of the primary transcript of miR-21^17^. Therefore, we assessed the role of STAT3 in BCR-induced miR-21 upsurge by pharmacological inhibition. For that we incubated Ramos transfectants with 5 µM JSI-124 for 1 hour and then stimulated the BCR and analyzed miR-21 expression. After 48 hours, we observed that the inhibition of STAT3 blocked the upregulation of miR-21 after BCR stimulation regardless the expression of ZAP-70 (Fig. 2e and f). Altogether these results indicate that the activation of the BCR signaling pathway, which is enhanced when ZAP-70 is expressed, is able to induce an increase in mature miR-21 via transcriptional activation of the primary transcript after a signaling pathway involving MAPK and STAT3 activation.

### The tumor suppressor genes PTEN, PDCD4 and PIAS3, targeted by miR-21, are downregulated after BCR stimulation

**Figure 3:**
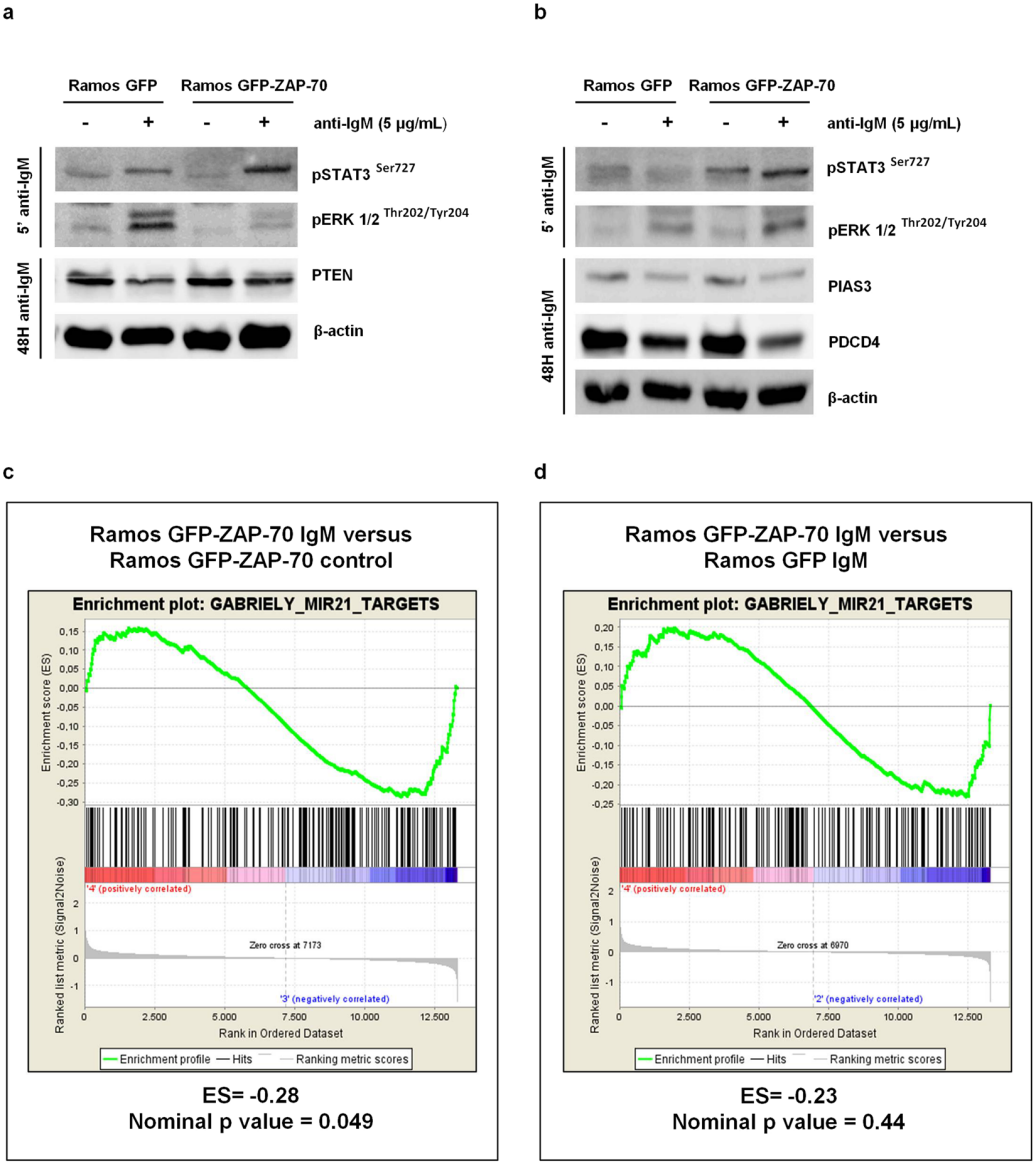
The tumor suppressor genes PTEN, PDCD4 and PIAS3, targeted by miR-21, are downregulated after BCR stimulation. (**a,b**) Ramos stable transfectants were stimulated with 5 µg/mL F(ab’)_2_ anti-IgM for 5 minutes and 48 hours. Enhanced STAT3 and ERK1/2 phosphorylation was observed in IgM-BCR stimulated cells for 5 minutes. PTEN, PIAS3 and PDCD4 downregulation was observed in IgM-BCR stimulated cells after 48 hours. (**c,d**) Gene set enrichment analysis (GSEA) for Gabriely_miR-21_targets gene set (N = 289). The panel shows GSEA of Ramos GFP-ZAP-70 stimulated with 5 µg/mL F(ab’)_2_ anti-IgM for 4 hours versus Ramos GPF-ZAP-70 unstimulated (**c**). The enrichment plot shows GSEA of Ramos GFP-ZAP-70 versus Ramos GFP cells, both stimulated with 5 µg/mL F(ab’)_2_ anti-IgM for 4 hours (**d**). ES: enrichment score.

MicroRNA miR-21 has been shown to target different genes, mostly involved in regulation of proliferation and survival^9^, including tumor suppressors such as PTEN^18^, PDCD4^19^ and PIAS3^20^. In order to assess if these genes were also modulated by miR-21 in malignant B cells, we stimulated the BCR of Ramos GFP and Ramos GFP-ZAP-70 cells and observed a modest downregulation at the protein level of PTEN, PDCD4 and PIAS3 (Fig. 3a and b). In addition, the global analysis of gene expression changes in Ramos GFP-ZAP-70 transfectants by Gene Set Enrichment Analysis (GSEA) showed a significant downregulation of miR-21 targets gene set after BCR stimulation (enrichment score: −0.28; nominal p-value: 0.049; Fig. 3c). The targets of miR-21 defined in the gene set where also downregulated when comparing Ramos GFP-ZAP-70 cells and Ramos GFP, both stimulated with anti-IgM, although the difference did not reach statistical significance (enrichment score: −0.23; nominal p-value: 0.44) (Fig. 3d).

### Co-culture of primary CLL cells with BMSC, CD40L and CpG ODN induces the expression of miR-21

**Figure 4:**
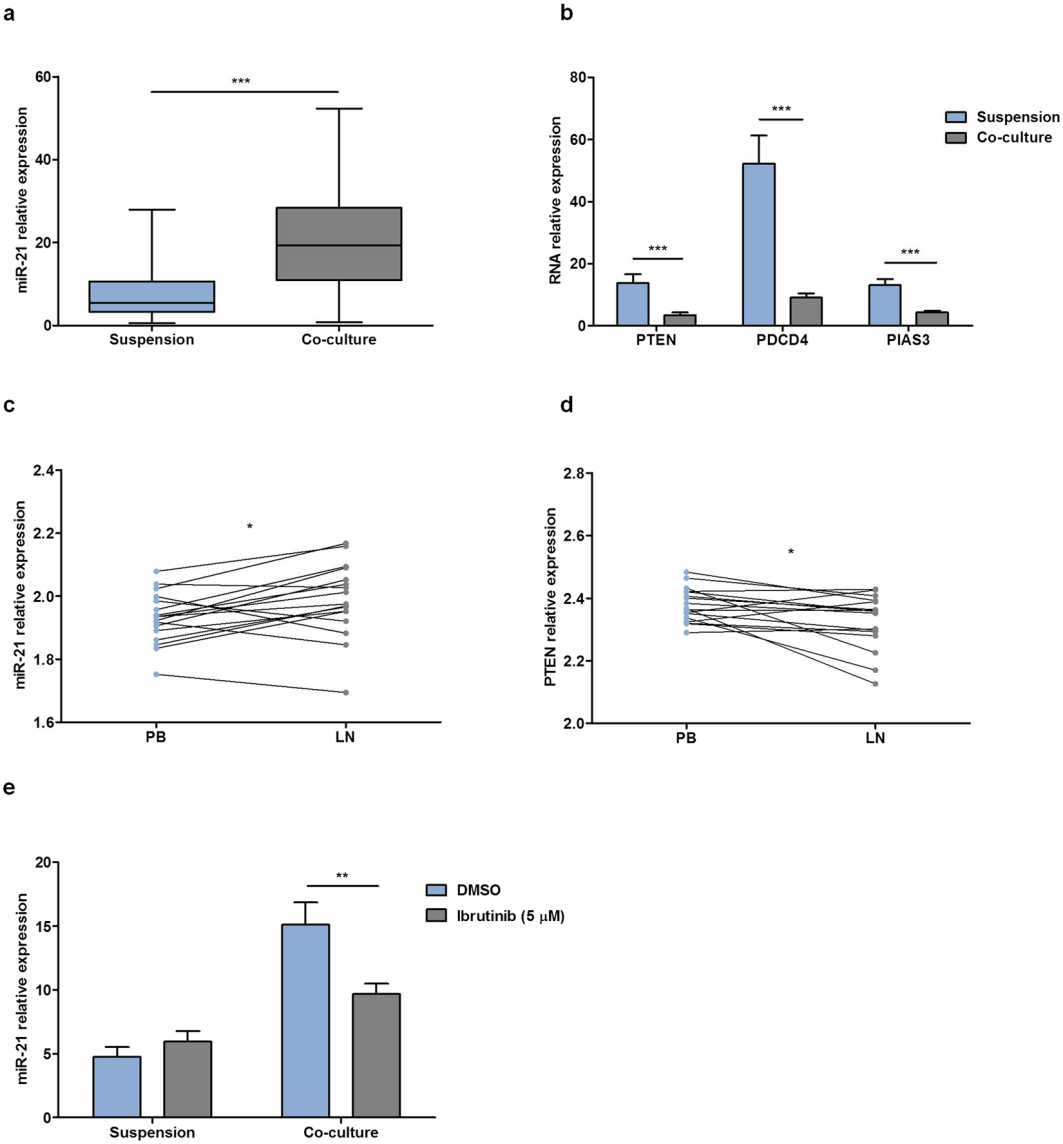
Co-culture of primary CLL cells with BMSC, CD40L and CpG ODN induces the expression of miR-21 and downregulation of the miR-21 targets PTEN, PDCD4 and PIAS3. (**a**) Primary CLL cells from 40 patients were cultured in suspension or in co-culture for 48 hours and miR-21 expression was measured by QRT-PCR. (**b**) Primary CLL cells from 22 patients were cultured in suspension or in co-culture for 48 hours. PTEN, PDCD4 and PIAS3 expression was determined by QRT-PCR. (**c**–**d**) Relative gene expression comparison between matched samples from peripheral blood (PB) and lymph nodes (LN) from 17 treatment-naive CLL patients, using public data from Y.Herishanu *et al*., Blood 2011^23^. mRNA expression level of miR-21 (**c**) and PTEN (**d**) were analyzed and compared by Wilcoxon matched pairs signed rank test. (**e**) PBMC from 13 patients were pre-treated with 5 µM PCI-32765 for 1 hour and cultured in suspension or in co-culture for 48 hours. Expression levels of miR-21 were measured by QRT-PCR. *P < 0.05, **P < 0.01, ***P < 0.0001.

With the aim of *in vitro* reproducing the microenvironment that CLL cells find in the proliferative centers *in vivo*, we have developed a co-culture system of primary CLL cells with the bone marrow stromal cell (BMSC) cell line UE6E7T-2, soluble CD40L, CpG ODN and anti-IgM which has been reported elsewhere^21^. Using this approach we observed an increase in survival, proliferation and chemoresistance in primary CLL cells. Interestingly, the co-culture also induced activation and modulation of expression of different molecules, including increased expression of ZAP-70^7^ and phosphorylation of ERK1/2 and STAT3^22^. Since we observed the involvement of the ZAP-70-ERK1/2-STAT3 axis in the induction of miR-21 expression in Ramos cells, we co-cultured primary CLL cells from 40 patients and observed a relative mean increase of 2.57 fold of miR-21 expression after 48 hours compared to the expression observed in cells kept in suspension (from 7.921 AU ± 1.091 to 20.35 AU ± 1.938; p < 0.0001) (Fig. 4a). This increase was accompanied by a concomitant downregulation of miR-21 targets PTEN, PDCD4 and PIAS3 at the mRNA level (PTEN from 13.76 AU ± 2.853 to 3.485 AU ± 0.878; PDCD4 from 52.3 AU ± 9.01 to 9.085 AU ± 1.371; PIAS3 from 13.07 AU ± 1.988 to 4.353 AU ± 0.569; p < 0.001 in all) (Fig. 4b). The herein described co-culture system is able to mimic the microenvironment found by primary CLL cells in lymph nodes, a tissue that shows constitutively active BCR signaling, as previously reported by Herishanu *et al*.^23^. Therefore, we interrogated the PubMed GEO database of expression data from paired samples of lymph nodes (LN), bone marrow (BM) and PB from patients with CLL for the differential expression of miR-21 and its targets in the different compartment. By paired Student’s t- test we observed that miR-21 expression level was significantly increased in LNs from patients with CLL compared to PB (from 1.931 ± 0.019 to 1.988 AU ± 0.028; p = 0.017) (Fig. 4c). Moreover, the expression of PTEN was lower in LN compared to PB (from 2.379 AU ± 0.013 to 2.325 AU ± 0.2; p = 0.019) (Fig. 4d), whereas PIAS3 and PDCD4 did not show any significant change (data not shown). Finally, we aimed to assess the role of the BCR signaling pathway in the induction of miR-21 in co-cultured primary CLL cells. For that we treated primary CLL cells from 13 patients with 5 µM BTK inhibitor ibrutinib for 1 hour before co-culturing them for 48 hours which significantly impaired miR-21 upregulation, in further agreement with the involvement of the BCR signaling pathway in the regulation of miR-21 expression in CLL (Fig. 4e).

### The increase in survival induced by co-culture of primary CLL cells with BMSC, CD40L and CpG ODN correlates with the upregulation of miR-21

**Figure 5:**
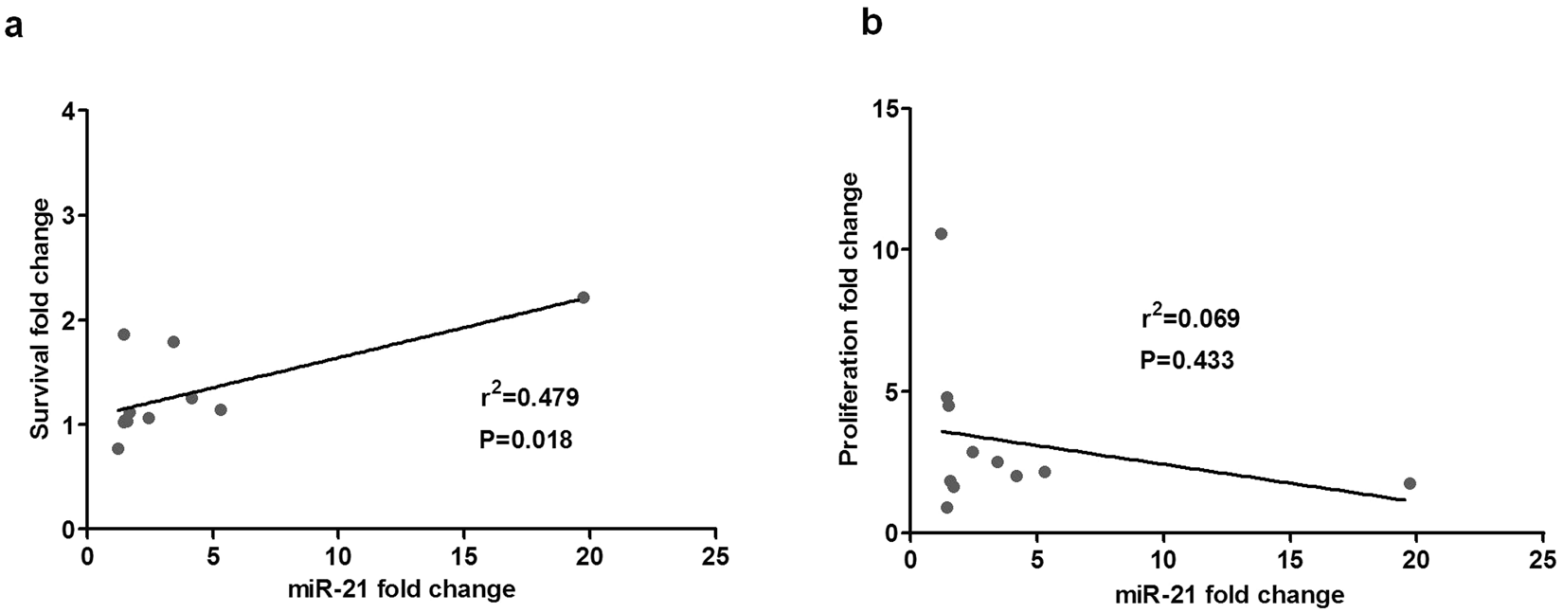
Co-culture of primary CLL cells with BMSC, CD40L and CpG ODN increases survival, proliferation and miR-21 expression. Survival and miR-21 increase are significantly and positively correlated. PBMC from 11 patients diagnosed with CLL were maintained in suspension or in co-culture for 48 hours. (**a**) Linear regression analysis of the correlation between fold changes of viability (viability in co-culture versus suspension) and fold changes of miR-21 mRNA expression (miR-21 expression in co-culture versus suspension). (**b**) Linear regression analysis of the correlation between fold changes of proliferation (proliferation in co-culture versus suspension) and fold changes of miR-21 mRNA expression (miR-21 expression in co-culture versus suspension). r^2^ is the coefficient of determination.

Finally, we aimed to study the implication of microRNA miR-21 in the induction of survival and proliferation by the microenvironment in primary CLL cells. For that, we co-cultured primary cells from 11 patients diagnosed with CLL for 48 hours in our co-culture system or in suspension and measured the increase in survival by means of annexinV-PI exclusion, and the proliferation by the percentage of Ki-67 positive CD19+/CD5+ CLL cells. As expected, the co-culture induced an increase in survival, proliferation, and the expression of miR-21 (Fig. 5). Interestingly, we also observed that the cytoprotection conferred by the microenvironment was significantly correlated to the fold change increase in miR-21 expression observed (r^2^ = 0.479, p = 0.018; Fig. 5a) indicating that miR-21 plays a role, among other factors, in that cytoprotection. The induction of proliferation, however, did not correlate with miR-21 increase (Fig. 5b).

## Discussion

The crosstalk between CLL cells and the microenvironment is able to strongly modulate biological processes in these cells, such as sustained survival, proliferation, circulation between different compartments, lack of response to apoptotic signals, and response to different therapeutic agents. CLL cells that express unmutated IGHV genes have an increased BCR signaling, partly because of the usual concomitant expression of ZAP-70 protein which directly enhances that signaling^24,25^. Moreover, CLL cells temporary residing in the LNs activate downstream elements of the BCR signaling pathway, regardless of the IGHV mutational status^23^. Therefore, we believe that contributing to further dissecting the different elements participating in this pathway is of relevance for the complete understanding of CLL intricate biology.

MicroRNAs have been described to be of relevance for CLL in different facets, nonetheless the first microRNAs to be proved to be able to act as tumor suppressors were miR-15a and miR-16-1, which are deleted in around 50% of cases of CLL^26^. Also, both overexpression or downregulation of different microRNAs have been related to features of aggressive disease, including miR-155^27^ and miR17-92^28^ whose expression is regulated by microenvironmental stimulus, rather than by the presence of genetic alterations. MicroRNA-21 was the second microRNA to be directly shown to cause a tumor phenotype in a mouse model, specifically the development of a pre-B lymphoproliferative disease, and thus being a genuine oncogene or *onco-miR* ^11^. Although not being extensively studied in CLL, its overexpression has been related to refractoriness to fludarabine^15^, to a shorter overall survival, and to a higher probability of progression^16^. Also, the cytoprotection offered by IL-4 correlated with miR-21 fold change in a *ex vivo* model of CLL^29^. Herein, we initially described how primary CLL cells with basal high expression of ZAP-70 also have higher levels of miR-21 expression, which prompted us to further study the potential role of ZAP-70 protein in the regulation of the expression of miR-21. However, although the mean expression of miR-21 was higher in the group of patients with high expression of ZAP-70, we did not observe a significant linear correlation between the percentage ZAP-70-positive CLL cells and the level of miR-21 expression as assessed by QRT-PCR (data not shown); therefore, even though ZAP-70 is probably directly involved in the regulation of the expression of miR-21 in primary CLL cells, it is likely that other proteins and signaling pathways are also participating in miR-21 regulation of expression in CLL, precluding the observation of a direct positive correlation between miR-21 and ZAP-70 expression in primary CLL cells. ZAP-70 protein has been directly involved in previous reports by us and others in the modulation of different proteins implicated in the crosstalk of CLL cells with the microenvironment, such as CCR7^24^ and CXCR4^6^. Using a system that we had previously described^24^, in which we study the role of ZAP-70 by transfecting mature neoplastic B cells, in this study we describe how ZAP-70 participates in the induction of miR-21 transcription by a pathway involving MAPK and STAT3 activation.

MicroRNAs can potentially target several different genes, mainly by binding to the 3′UTR region of mRNA and controlling mRNA stability and/or translation^30^; in the particular case of miR-21, the majority of its described targets are tumor suppressor genes involved in proliferation, apoptosis or inflammation^9,31^. For the current study we chose to evaluate the three miR-21 target genes, namely PTEN, PDCD4 and PIAS3, defined to be relevant for B-cell malignancies. In Ramos expressing ZAP-70 a slight but reproducible downregulation of PTEN, PDCD4 and PIAS3 at the protein level after BCR activation was detected. In the case of primary CLL cells that were co-cultured in conditions mimicking the microenvironment, there was an upregulation of miR-21 expression that could be explained in part by the concomitant increase of ZAP-70, while other signals from the microenvironment are probably involved in the upregulation of miR-21. In this sense, the stimulation of CLL cells with IL-4 has also been shown to induce upregulation of miR-21^29^. The co-culture of CLL cells herein described therefore induced the upregulation of miR-21 and a subsequent clear downregulation at the mRNA level of PTEN, PDCD4 and PIAS3, pointing towards the direct downmodulation by miR-21 but not ruling out the possibility of other microenvironmental factors involved in the regulation of these tumor suppressors. Remarkably, the increase of miR-21 in co-cultured CLL cells did actually correlate with the increase in survival or protection from spontaneous apoptosis provided by the microenvironment, again suggesting a clear role for miR-21 and its targets in this phenomenon. The upregulation of the BCR pathway that can be induced by ZAP-70 protein is one of the differential features observed when comparing paired samples of CLL cells residing in the peripheral blood or in the lymph nodes, where the signature of genes related to BCR signaling has been observed to be upregulated. Herein, we interrogated the publicly available data on GEP of paired samples from LNs and PB from patients with CLL provided by Herishanu and collaborators^23^ and observed that miR-21 was indeed significantly upregulated in LNs according to paired Wilcoxon t-test. The specific analysis of microRNAs differentially expressed between LNs and PB in CLL has been recently published by Saleh and collaborators^14^. They observe that miR-21 is 10 times higher in CLL compared to normal B cells, and also that the expression is higher in LNs compared to PB, although the difference did not reach statistical significance. In order to further relate the increase of miR-21 that we observed after stimulating primary CLL cells with microenviromental stimuli with BCR signaling we inhibited this pathway with the widely used BTK inhibitor ibrutinib, which caused a marked impairment in the upregulation of miR-21. In conclusion, we have shown that stimuli from the microenvironment are capable of regulating the expression of miR-21 and the tumor suppressor genes (PTEN, PDCD4 and PIAS3) in CLL via a signaling pathway involving ZAP-70, MAPK and STAT3 transcription factor which correlates with the induction of survival by the microenvironment. Although further experiments are warranted in order to fully elucidate other factors apart from ZAP-70 involved in the regulation and functional role of miR-21 in CLL, these results help to enlighten the biology behind the adverse clinical outcome of patients with CLL having high expression of ZAP-70 and enhanced activation of the BCR signaling pathway.

## Methods

### Primary CLL cells

Peripheral blood mononuclear cells (PBMC) from 77 treatment-naïve patients diagnosed with CLL were isolated by Ficoll-Paque Plus (GE Healthcare) density gradient centrifugation and cryopreserved in liquid nitrogen until analysis. Patients were selected on the basis of availability of frozen samples for biological studies. Written informed consent was obtained from all patients in accordance with the Declaration of Helsinki and all experiments were conducted in accordance to such declaration. The study was approved by the clinical investigation ethical committee from Vall d’Hebron University Hospital (Barcelona, Spain). ZAP-70 expression was determined by flow cytometry: first, the expression of cell surface antigens was detected using the following fluorochrome-labeled antibodies: CD19-ECD, CD5-PC5.5 (Beckman Coulter) and CD3-PE-Cy7 (Beckton Dickinson) followed by intracellular detection of ZAP-70 using the IntraSure kit (Beckton Dickinson) and primary antibody anti-ZAP-70-PE (Beckman Coulter). Cases with equal or more than 20% of ZAP-70-positive cells, using autologous T cells as an internal control, were considered to be ZAP-70 positive, as previously described^4^.

For determination of miR-21 expression according to ZAP-70 expression, CD19-positive CLL cells from 33 cases were purified prior to nucleic acid extraction by positive selection using anti-CD19 magnetic microbeads (Miltenyi Biotech). Purity of CD19+ cells greater than 95% was obtained in all samples. For the experiments in Figs 4 and 5, additional 44 primary samples from patients with CLL were analyzed. Patient characteristics are summarized in Table 1.

### Cell lines

Cell lines were cultured in RPMI-1640 medium (Gibco®) supplemented with 10% fetal bovine serum (FBS), 50 µg/mL penicillin/streptomycin and 2 mM L-glutamine at 37 °C in a humidified atmosphere (5% CO_2_). Ramos cell line derives from a Burkitt’s lymphoma and it was obtained from American Type Culture Collection (ATCC). Ramos cells were stably transfected with a vector encoding for ZAP-70 protein fused with Green fluorescent protein (GFP) or GFP only as a control^24^. Jurkat cells are derived from a T-cell acute lymphoblastic leukemia and they were obtained from ATCC. Jurkat cells were stimulated with pervanadate (3 mM H_2_O_2_/1 mM NaVO_4_) for 5 minutes at 37 °C, as a positive control for phosphoproteins. Human Bone Marrow Stromal Cell (BMSC) cell line UE6E7T-2 was provided by Riken Cell Bank (Ibaraki, Japan) and cultured at 37 °C in 5% CO_2_ atmosphere in Dulbecco’s Modified Eagle Medium (DMEM; Gibco®) supplemented with 2 mM L-glutamine, 10% heat-inactivated FBS and 50 µg/mL penicillin/streptomycin.

### Co-culture of primary CLL cells

PBMC from patients diagnosed with CLL were co-cultured with BMSC at a 100:1 ratio and stimulated with 1 µg/mL CD40L (Peprotech) and 1.5 µg/mL CpG ODN (ODN2006; Invivogen). After 48 hours, CLL cells were harvested by gently washing off, leaving the adherent stromal cell layer intact.

### Quantitative RT-PCR

Total RNA from cell lines and PBMC from patients was extracted by using TRIzol reagent (Invitrogen). For miRNA amplification, RNA was reverse transcribed into single stranded complementary DNA (cDNA) using ±TaqMan MicroRNA Assays system (Applied Biosystems) and specific primer for hsa-miR-21 and RNU6B as control. mRNA was reverse transcribed into cDNA using M-MLV Reverse Transcriptase (Invitrogen) according to the manufacturer’s instructions.

Both mRNA and miRNA levels were detected by either TaqMan Assays (PTEN Hs02621230_s1, PDCD4 Hs00377253_m1, PIAS3 Hs00966035_m1) or TaqMan MicroRNA Assay (hsa-miR-21 000397) (Applied Biosystems). RNU6B (001093) and GAPDH (Hs03929097_g1) were used as internal controls for miRNA and mRNAs, respectively. Primary miR-21 was amplified with specific primers previously described (see ref.^17^) and quantified using SYBR GreenER qPCR SuperMix. QRT-PCR was performed in an ABI Prism 7900HT Sequence Detector System (Applied Biosystems) in triplicates for each cDNA. ABI SDS 2.4 Software (Life Technologies) was used to calculate relative expression, using comparative Ct method (ΔΔCt). RNA expression levels are defined as arbitrary units (AU) using Ramos cell line as a calibrator.

### Immunobloting

BCR was stimulated with 5 µg/mL F(ab)_2_ anti-IgM (Invitrogen). ERK1/2 and Akt were inhibited with PD98059 (Cell Signaling Technology, Inc.) and LY294002 (Sigma-Aldrich), respectively. Protein from cell lines was extracted using 20 mM Tris pH 7.4, 1 mM EDTA, 140 mM NaCl, 1% of NP-40 supplemented with 2 mM sodium vanadate and protease inhibitor cocktail (Sigma-Aldrich) for 30 minutes at 4 °C. Protein concentration was determined using the Bio Rad Protein Assay system (Bio-Rad). Equal amounts of denatured protein were separated by 10% poliacrilamide-SDS gel electrophoresis. The size-separated proteins were transferred to Immobilon-phosphate membranes (Millipore). Membranes were blocked with 5% milk/TBS-T (Tris-Buffered Saline Tween-20) for 1 hour at room temperature (RT). Membranes were incubated overnight at 4 °C with primary antibodies against phospho-ZAP-70^Tyr319^/Syk^Tyr352^, phospho-Akt^Ser473^, phospho-ERK1/2^Thr202/Tyr204^, phospho-STAT3^Ser727^, Akt, ERK1/2, PTEN and PDCD4 (Cell signalling Technology Inc.), ZAP-70 (clone 2F3.2; Upstate Biotechnology), PIAS3 (WuXi AppTec.ABGENT) and β-actin (Abcam). Immunodetection was done with the corresponding IgG HRP-linked secondary antibodies (Dako North America) for 1 hour at RT. Chemiluminescent images were obtained using LAS-4000 equipment (Buckinghamshire).

### Cell viability and proliferation

Cell viability was evaluated by surface annexin V-fluorescein isothiocynate (FITC) and propidium iodide (PI) double-negative staining assessed by flow cytometry (Bender MedSystems).

Expression of Ki-67 was used to measure cell proliferation rate by flow cytometry. Surface staining of 1 × 10^6^ cells was performed with the following monoclonal antibodies conjugated to a fluorochrome: CD19-phycoerythrin (PE) and CD5-allophycocyanine (APC). Intracellular staining was performed using FITC-labeled antibody against Ki-67 (Becton Dickinson) after fixation and permeabilization using the BD Intrasure kit (Becton Dickinson) following the manufacturer’s instructions. Cells were acquired in a Navios^TM^ cytometer and the results were analyzed using the FCS Express 4 software (De Novo Software).

### Statistical analysis

Results are shown as the mean ± standard error of the mean (SEM) of values obtained in three or more independent experiments. Statistical analysis between paired samples was performed using Student’s paired two-tailed t- test or Wilcoxon matched pairs signed rank test, difference between groups was analyzed using the Mann–Whitney U test, and P < 0.05 was considered significant. Gene set enrichment analysis (GSEA) was performed using the Gabriely_miR-21_targets gene set from the Molecular Signatures Data Base in the GSEA software from the Broad Institute^32,33^. Linear regression analysis (Spearman’s rank  correlation) was used to study the correlation between miR-21 expression and survival or proliferation. Analyses were performed using the biostatistics software package SPSS Version 17 (IBM). Results were graphed with GraphPad Prism software Version 5.0.

### Data Availability

The data that support the findings of this study are available from the corresponding author on reasonable request.

## References

[1] ten Hacken E, Burger JA (2014). Molecular pathways: targeting the microenvironment in chronic lymphocytic leukemia–focus on the B-cell receptor. Clin. Cancer Res. Off. J. Am. Assoc. Cancer Res..

[2] Byrd JC (2015). Three-year follow-up of treatment-naïve and previously treated patients with CLL and SLL receiving single-agent ibrutinib. Blood.

[3] Damle RN (1999). Ig V gene mutation status and CD38 expression as novel prognostic indicators in chronic lymphocytic leukemia. Blood.

[4] Crespo M (2003). ZAP-70 expression as a surrogate for immunoglobulin-variable-region mutations in chronic lymphocytic leukemia. N. Engl. J. Med..

[5] Sivina M (2011). CCL3 (MIP-1α) plasma levels and the risk for disease progression in chronic lymphocytic leukemia. Blood.

[6] Calpe E (2013). ZAP-70 promotes the infiltration of malignant B-lymphocytes into the bone marrow by enhancing signaling and migration after CXCR4 stimulation. PloS One.

[7] Purroy, N. *et al*. Co-culture of primary CLL cells with bone marrow mesenchymal cells, CD40 ligand and CpG ODN promotes proliferation of chemoresistant CLL cells phenotypically comparable to those proliferating *in vivo*. *Oncotarget* (2014). 10.18632/oncotarget.2939 PMC4480705 25544766

[8] Bottoni A, Calin GA (2014). MicroRNAs as Main Players in the Pathogenesis of Chronic Lymphocytic Leukemia. MicroRNA Shāriqah United Arab Emir..

[9] Jazbutyte V, Thum T (2010). MicroRNA-21: from cancer to cardiovascular disease. Curr. Drug Targets.

[10] Fu X (2011). Prognostic role of microRNA-21 in various carcinomas: a systematic review and meta-analysis. Eur. J. Clin. Invest..

[11] Medina PP, Nolde M, Slack FJ (2010). OncomiR addiction in an *in vivo* model of microRNA-21-induced pre-B-cell lymphoma. Nature.

[12] Thapa DR (2012). B-cell activation induced microRNA-21 is elevated in circulating B cells preceding the diagnosis of AIDS-related non-Hodgkin lymphomas. AIDS Lond. Engl..

[13] Calin GA (2005). A MicroRNA signature associated with prognosis and progression in chronic lymphocytic leukemia. N. Engl. J. Med..

[14] Saleh LM (2016). Ibrutinib downregulates a subset of miRNA leading to upregulation of tumor suppressors and inhibition of cell proliferation in chronic lymphocytic leukemia. Leukemia.

[15] Ferracin M (2010). MicroRNAs involvement in fludarabine refractory chronic lymphocytic leukemia. Mol. Cancer.

[16] Rossi S (2010). microRNA fingerprinting of CLL patients with chromosome 17p deletion identify a miR-21 score that stratifies early survival. Blood.

[17] Löffler, D. Interleukin-6 dependent survival of multiple myeloma cells involves the Stat3-mediated induction of microRNA-21 through a highly conserved enhancer. *Blood* **110**, 1330–1333 (2007). 10.1182/blood-2007-03-081133 17496199

[18] Wickramasinghe NS (2009). Estradiol downregulates miR-21 expression and increases miR-21 target gene expression in MCF-7 breast cancer cells. Nucleic Acids Res..

[19] Frankel LB (2008). Programmed cell death 4 (PDCD4) is an important functional target of the microRNA miR-21 in breast cancer cells. J. Biol. Chem..

[20] Xiong Q (2012). Identification of novel miR-21 target proteins in multiple myeloma cells by quantitative proteomics. J. Proteome Res..

[21] Purroy, N. *et al*. Targeting the proliferative and chemoresistant compartment in chronic lymphocytic leukemia by inhibiting survivin protein. *Leukemia*, doi:10.1038/leu.2014.96 (2014). 10.1038/leu.2014.96 24618734

[22] Purroy N (2017). Inhibition of BCR signaling using the Syk inhibitor TAK-659 prevents stroma-mediated signaling in chronic lymphocytic leukemia cells. Oncotarget.

[23] Herishanu Y (2011). The lymph node microenvironment promotes B-cell receptor signaling, NF-kappaB activation, and tumor proliferation in chronic lymphocytic leukemia. Blood.

[24] Calpe E (2011). ZAP-70 enhances migration of malignant B lymphocytes toward CCL21 by inducing CCR7 expression via IgM-ERK1/2 activation. Blood.

[25] Chen L (2008). ZAP-70 enhances IgM signaling independent of its kinase activity in chronic lymphocytic leukemia. Blood.

[26] Klein U (2010). The DLEU2/miR-15a/16-1 cluster controls B cell proliferation and its deletion leads to chronic lymphocytic leukemia. Cancer Cell.

[27] Cui B (2014). MicroRNA-155 influences B-cell receptor signaling and associates with aggressive disease in chronic lymphocytic leukemia. Blood.

[28] Bomben R (2012). The miR-17∼92 family regulates the response to Toll-like receptor 9 triggering of CLL cells with unmutated IGHV genes. Leukemia.

[29] Ruiz-Lafuente N (2015). IL-4 Up-Regulates MiR-21 and the MiRNAs Hosted in the CLCN5 Gene in Chronic Lymphocytic Leukemia. PloS One.

[30] Liu J (2008). Control of protein synthesis and mRNA degradation by microRNAs. Curr. Opin. Cell Biol..

[31] Buscaglia LEB, Li Y (2011). Apoptosis and the target genes of microRNA-21. Chin. J. Cancer.

[32] Subramanian A (2005). Gene set enrichment analysis: a knowledge-based approach for interpreting genome-wide expression profiles. Proc. Natl. Acad. Sci. USA..

[33] Mootha VK (2003). PGC-1alpha-responsive genes involved in oxidative phosphorylation are coordinately downregulated in human diabetes. Nat. Genet..

